# Supplementary figures and images for: CUT&RUN detects distinct DNA footprints of RNA polymerase II near the transcription start sites
Source: Chromosome Res. 2020 Oct 18;28(3):381–93. doi: 10.1007/s10577-020-09643-0 (PMC7691310; doi:10.1007/s10577-020-09643-0)

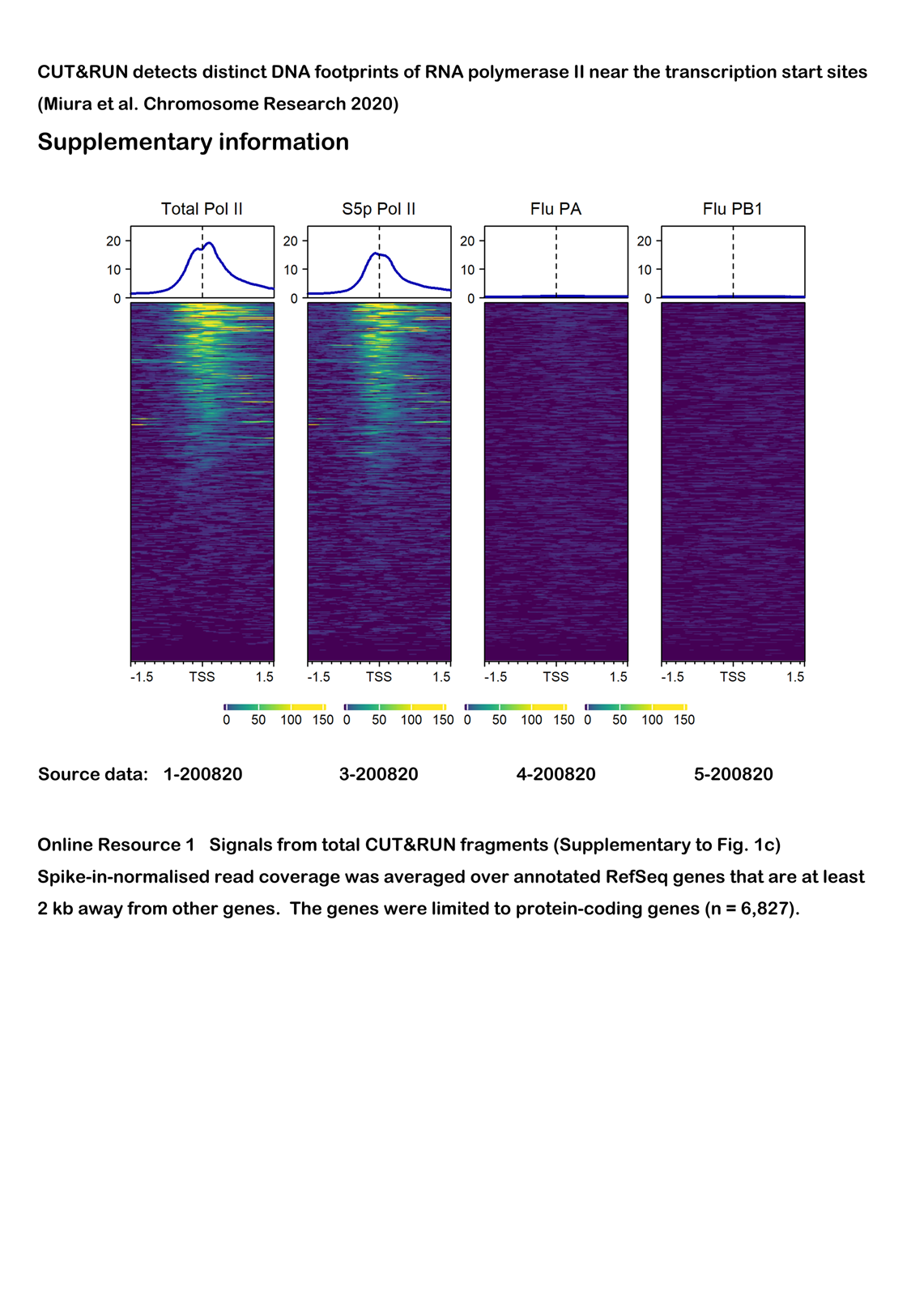

Supplement: Supplementary file 1 — Signals from total CUT&RUN fragments (Supplementary to Fig. 1c) (PNG 447 kb) [file 10577_2020_9643_Fig6_ESM.png]

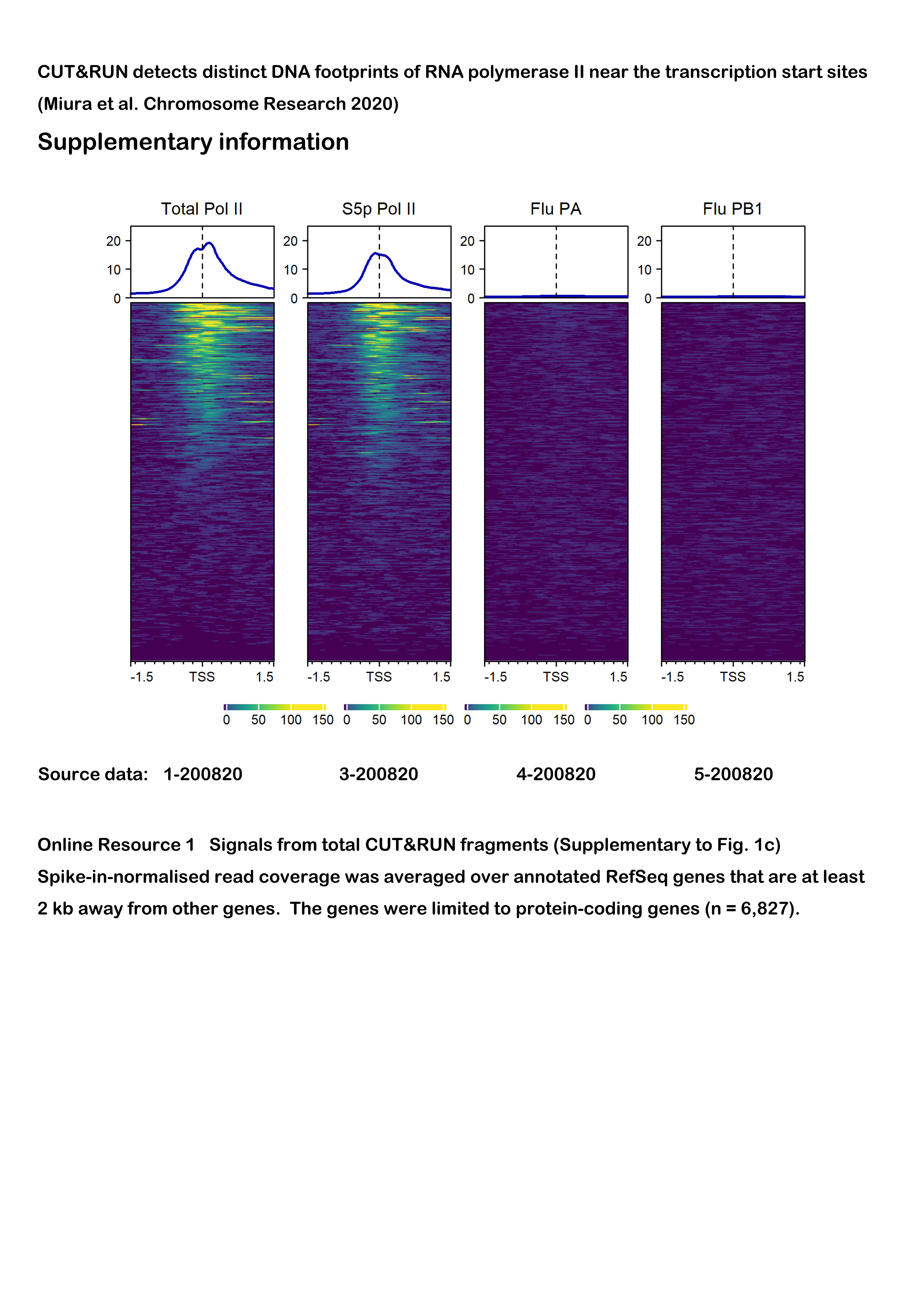

Supplement: Supplementary file 2 — High Resolution Image (TIF 4.16 mb) [file 10577_2020_9643_MOESM1_ESM.tif]

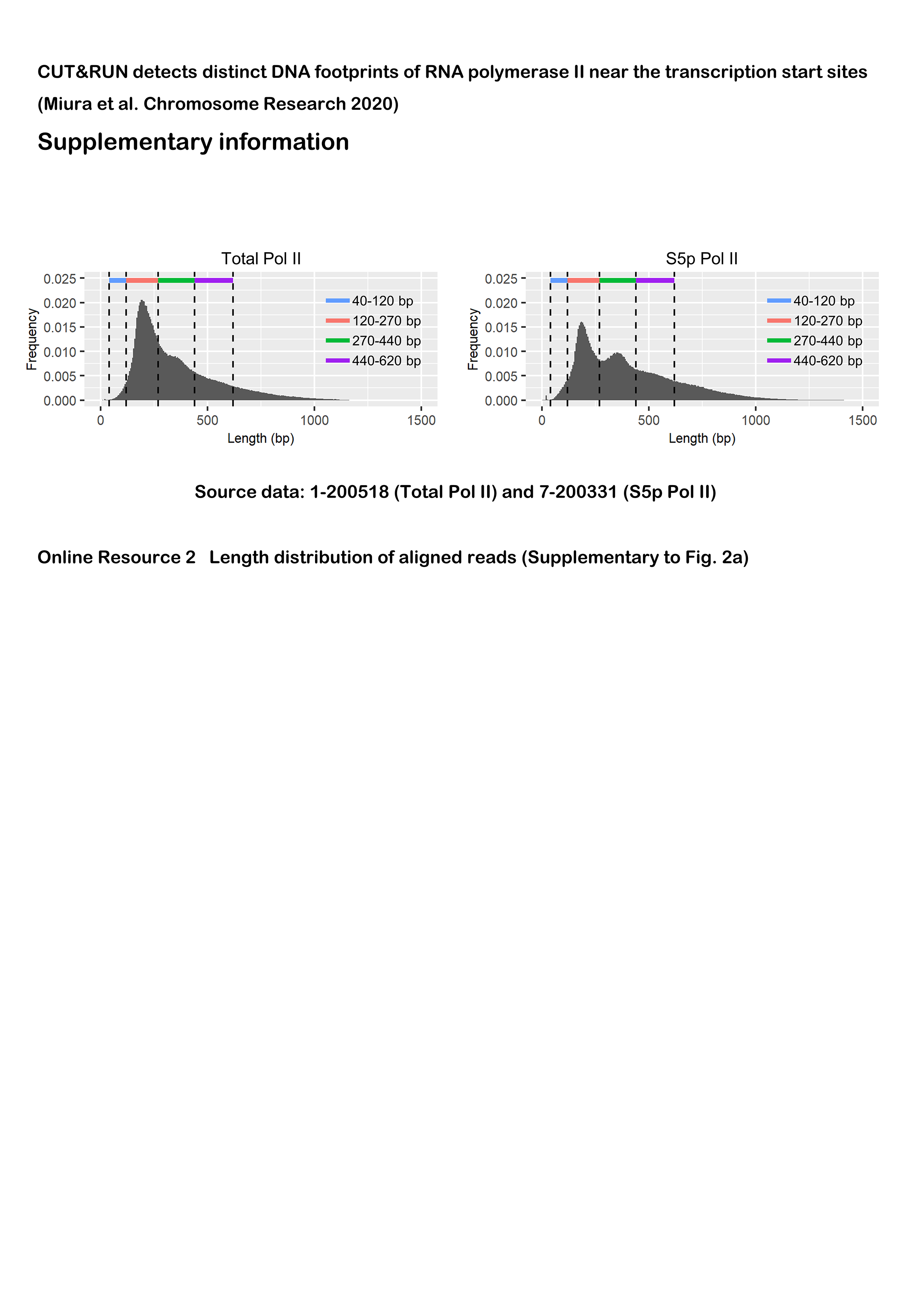

Supplement: Supplementary file 3 — Length distribution of aligned reads (Supplementary to Fig. 2a) (PNG 314 kb) [file 10577_2020_9643_Fig7_ESM.png]

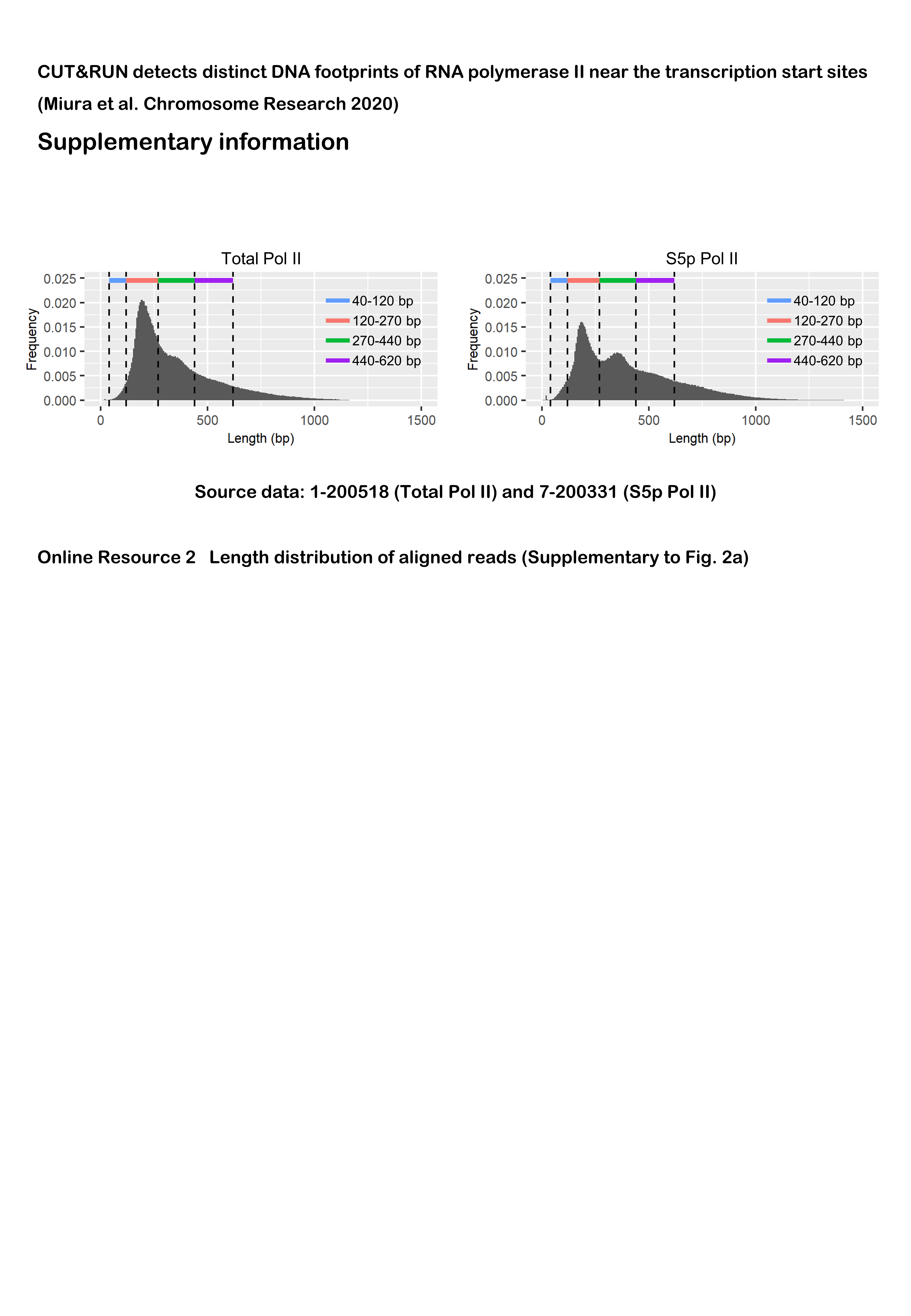

Supplement: Supplementary file 4 — High Resolution Image (TIF 1933 kb) [file 10577_2020_9643_MOESM2_ESM.tif]

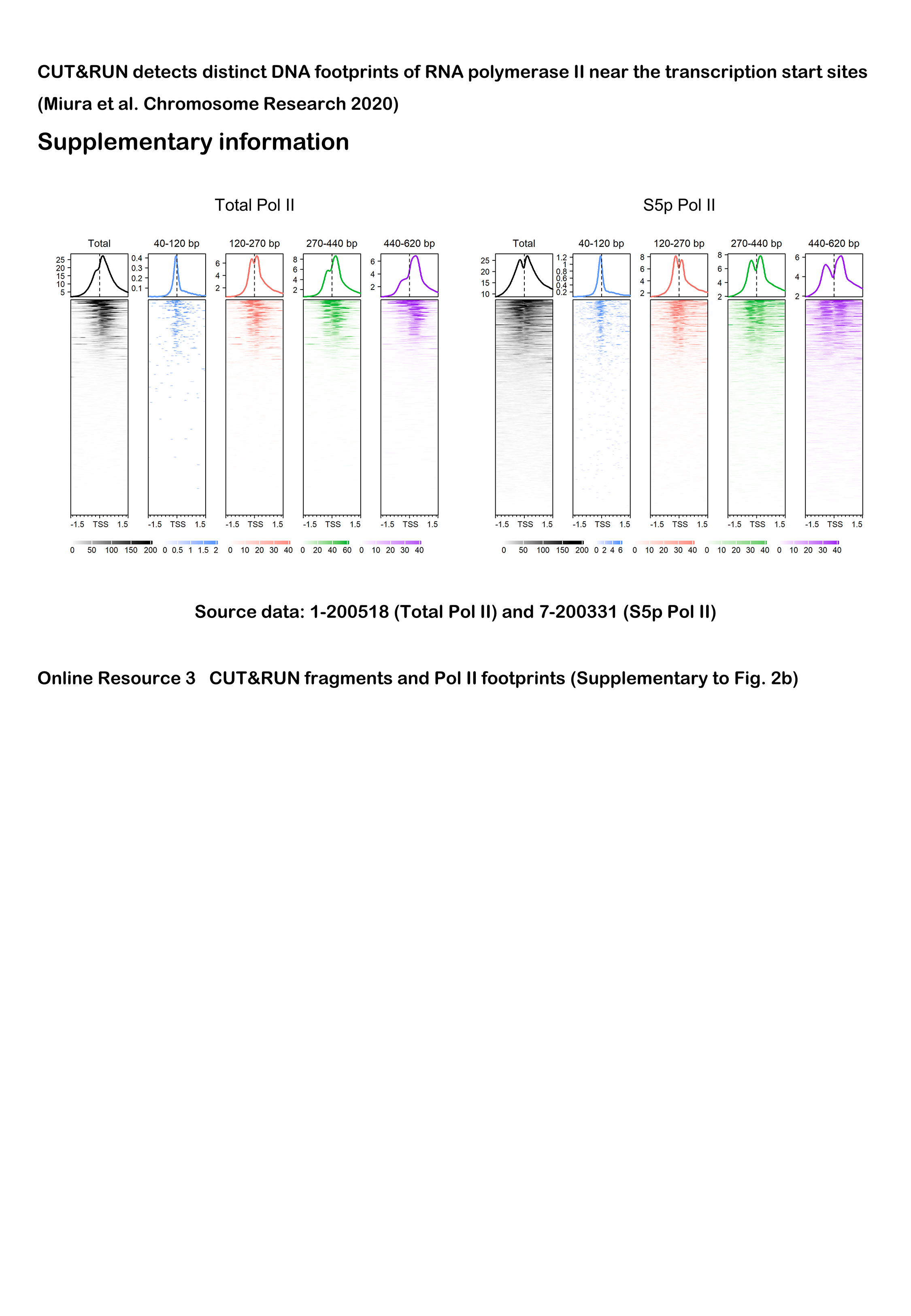

Supplement: Supplementary file 5 — CUT&RUN fragments and Pol II footprints (Supplementary to Fig. 2b) (PNG 847 kb) [file 10577_2020_9643_Fig8_ESM.png]

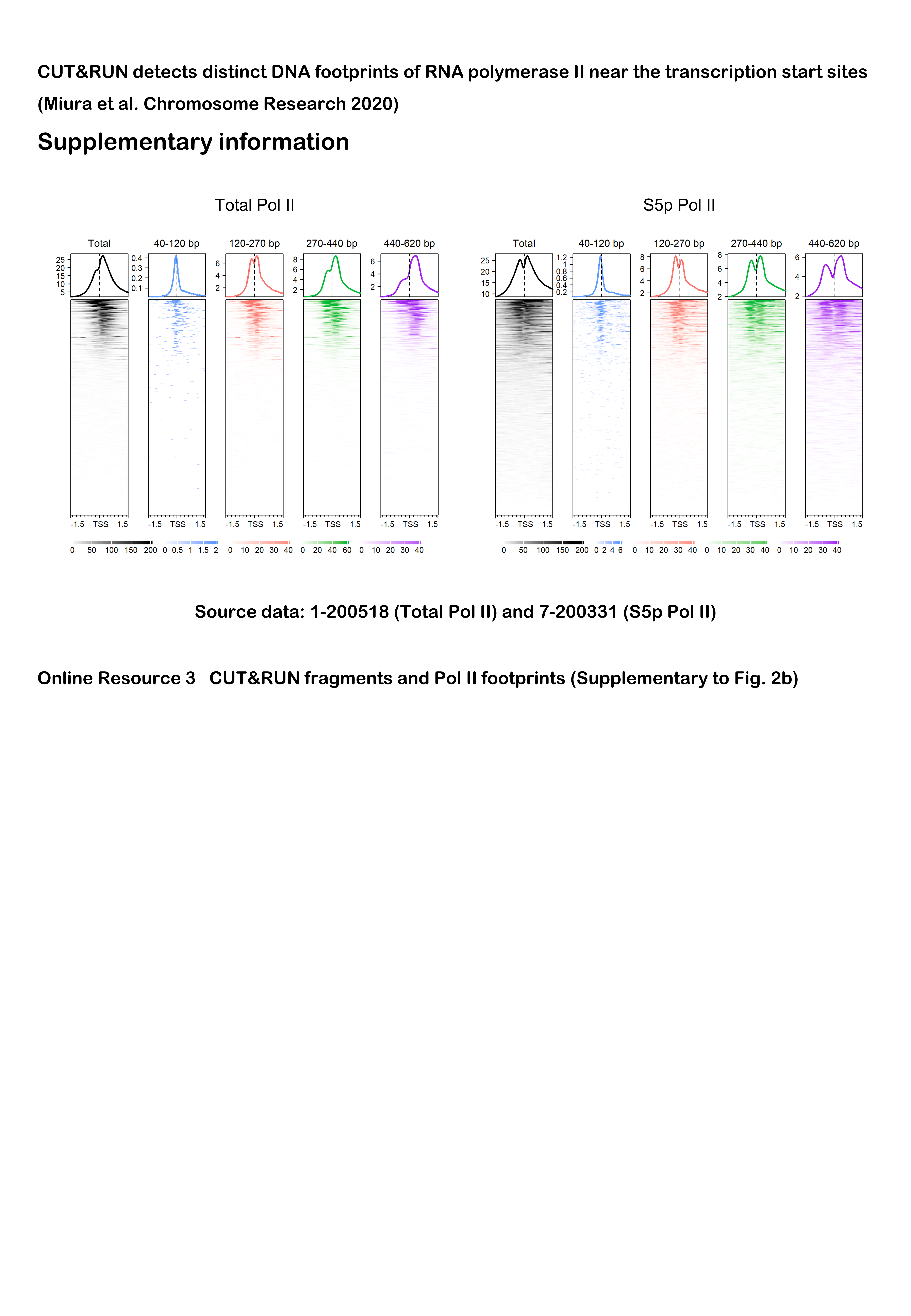

Supplement: Supplementary file 6 — High Resolution Image (TIF 3296 kb) [file 10577_2020_9643_MOESM3_ESM.tif]

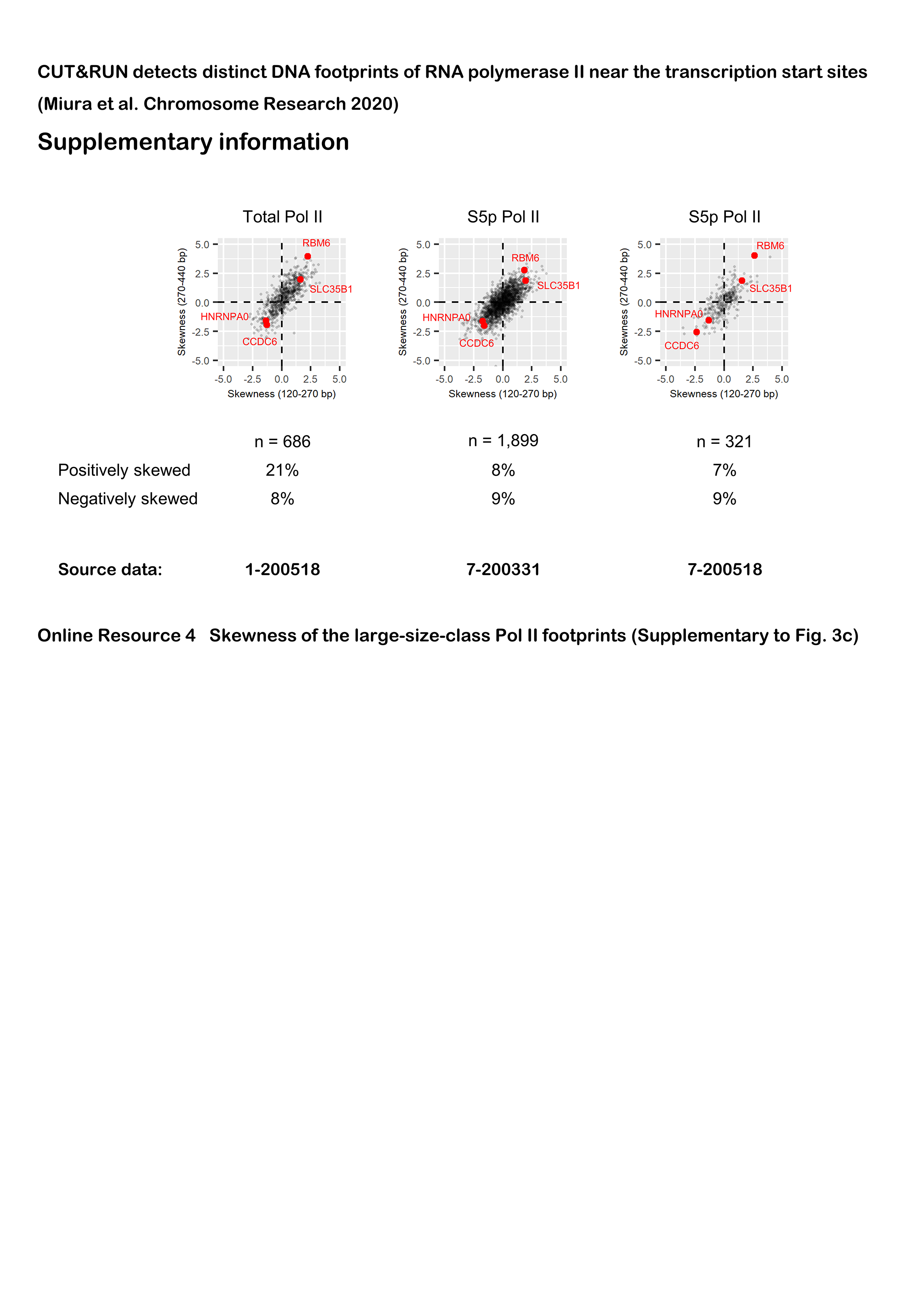

Supplement: Supplementary file 7 — Skewness of the large-size-class Pol II footprints (Supplementary to Fig. 3c) (PNG 449 kb) [file 10577_2020_9643_Fig9_ESM.png]

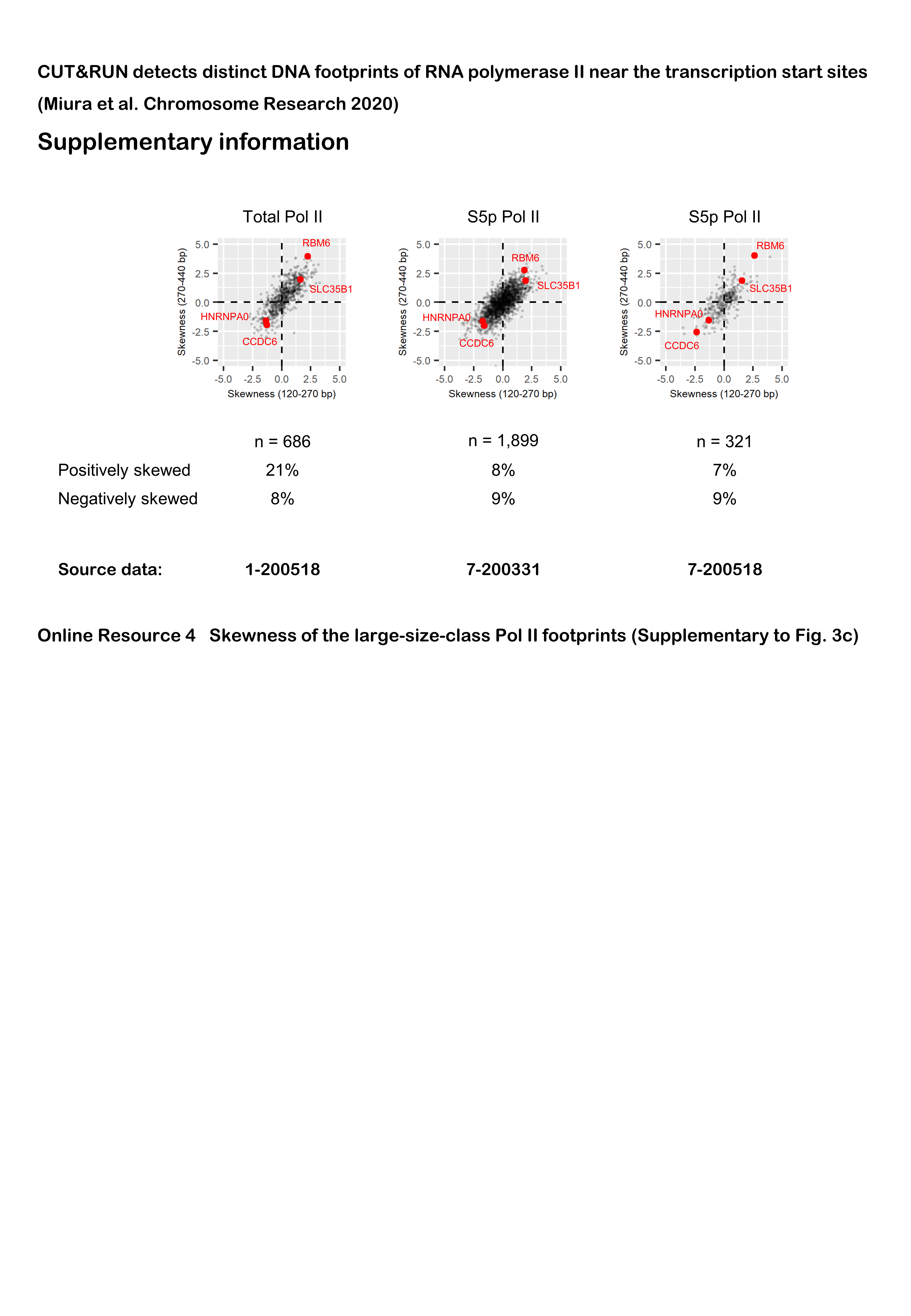

Supplement: Supplementary file 8 — High Resolution Image (TIF 2190 kb) [file 10577_2020_9643_MOESM4_ESM.tif]

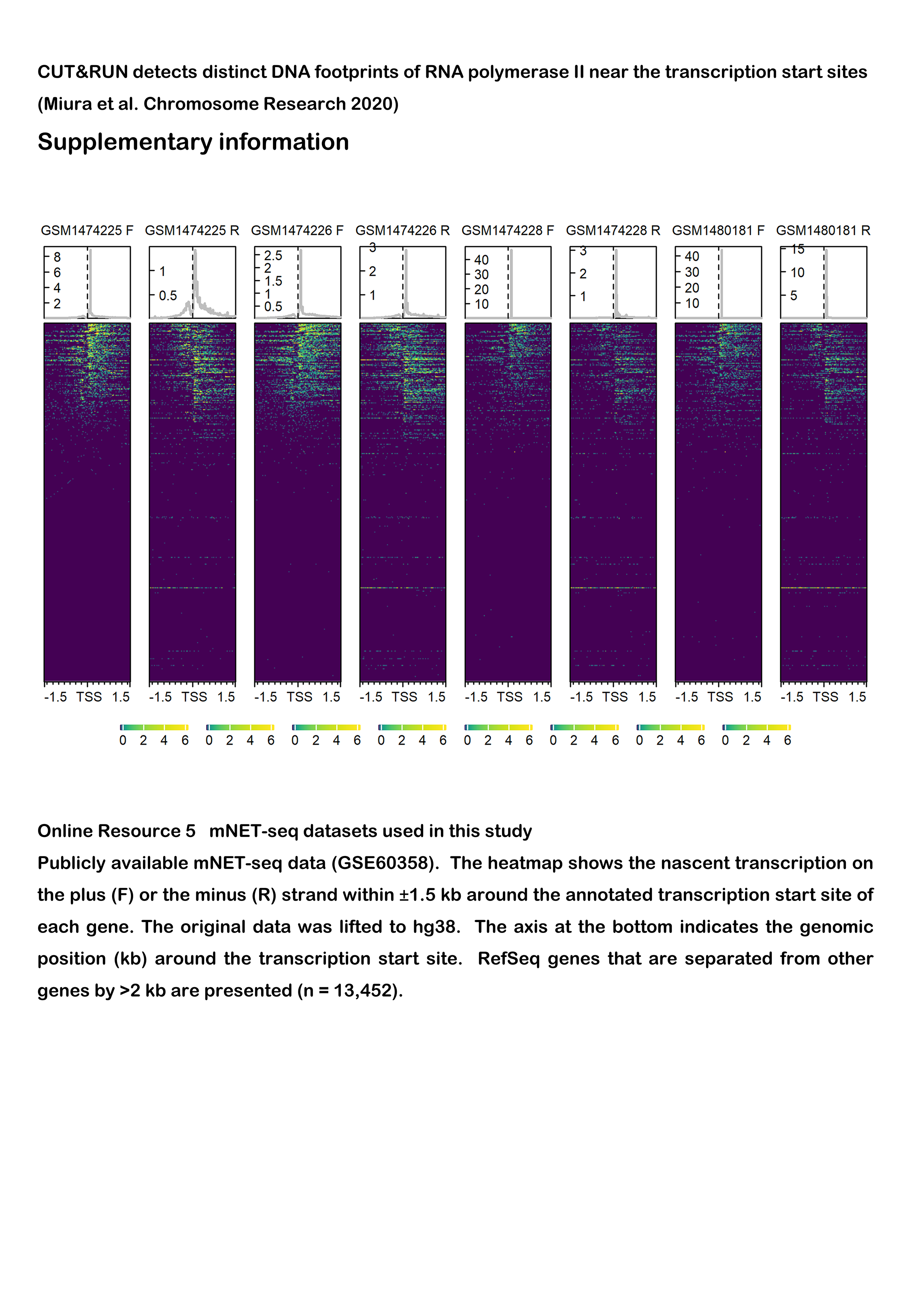

Supplement: Supplementary file 9 — mNET-seq datasets used in this study (PNG 1304 kb) [file 10577_2020_9643_Fig10_ESM.png]

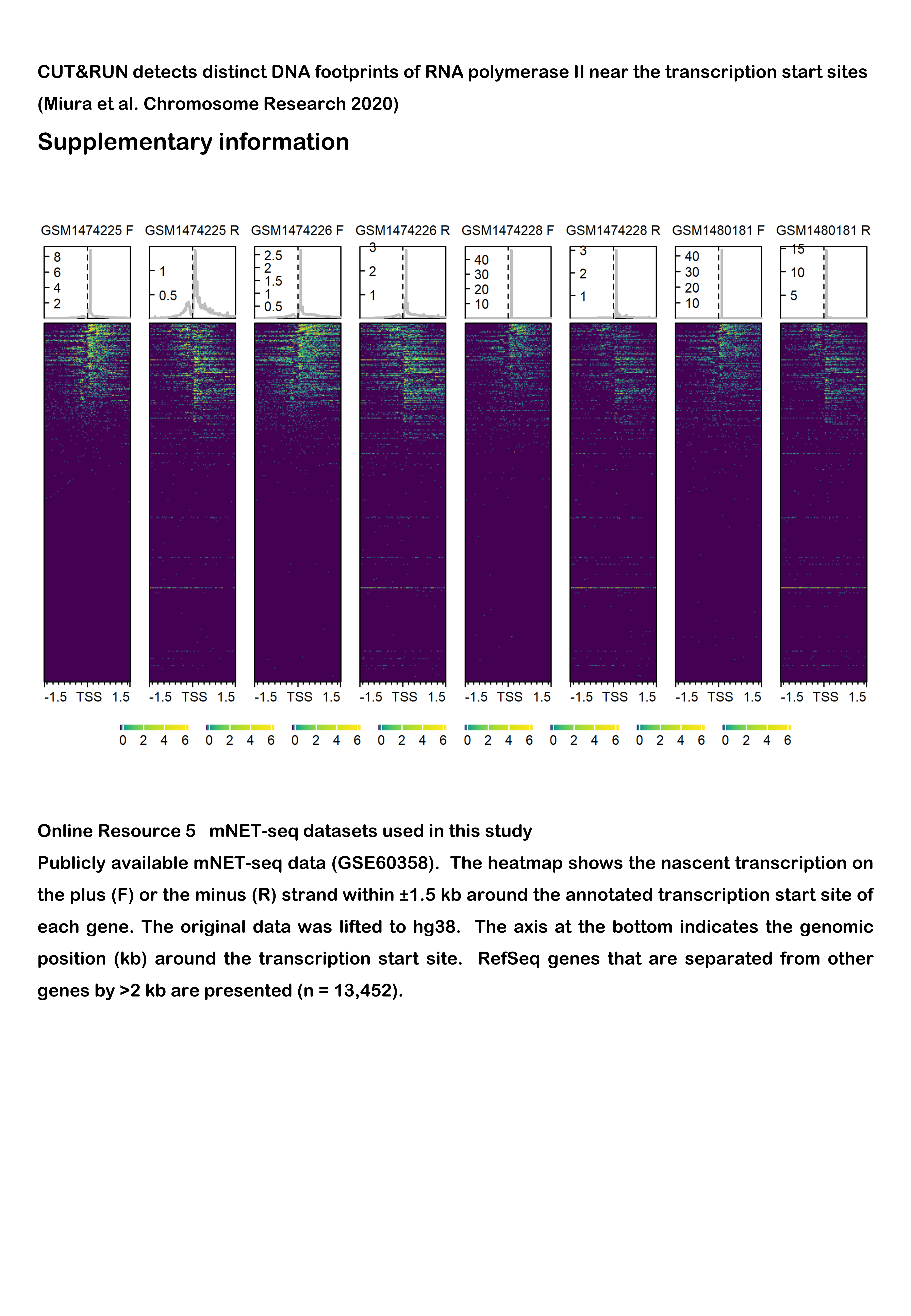

Supplement: Supplementary file 10 — High Resolution Image (TIF 5181 kb) [file 10577_2020_9643_MOESM5_ESM.tif]

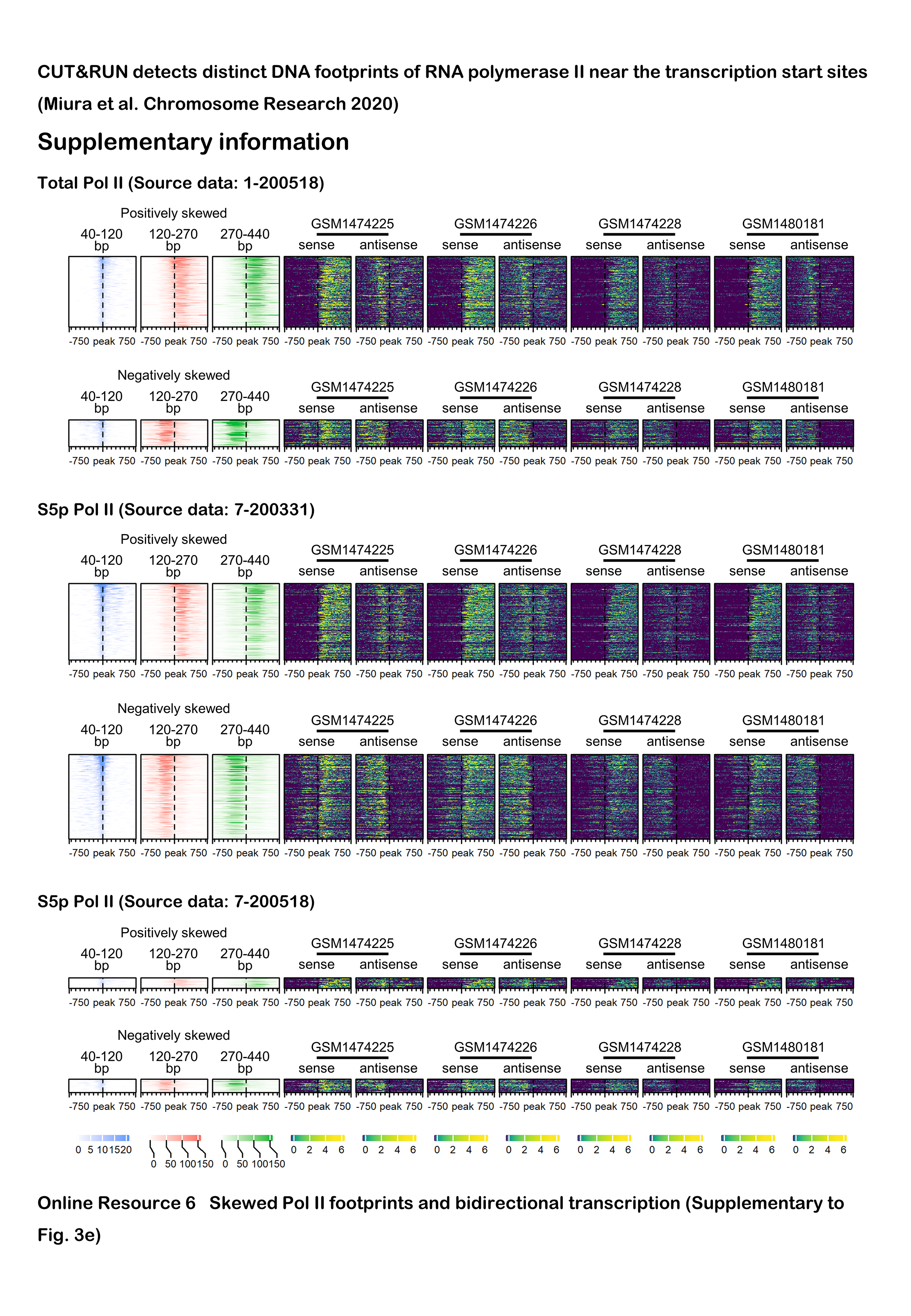

Supplement: Supplementary file 11 — Skewed Pol II footprints and bidirectional transcription (Supplementary to Fig. 3e) (PNG 3219 kb) [file 10577_2020_9643_Fig11_ESM.png]

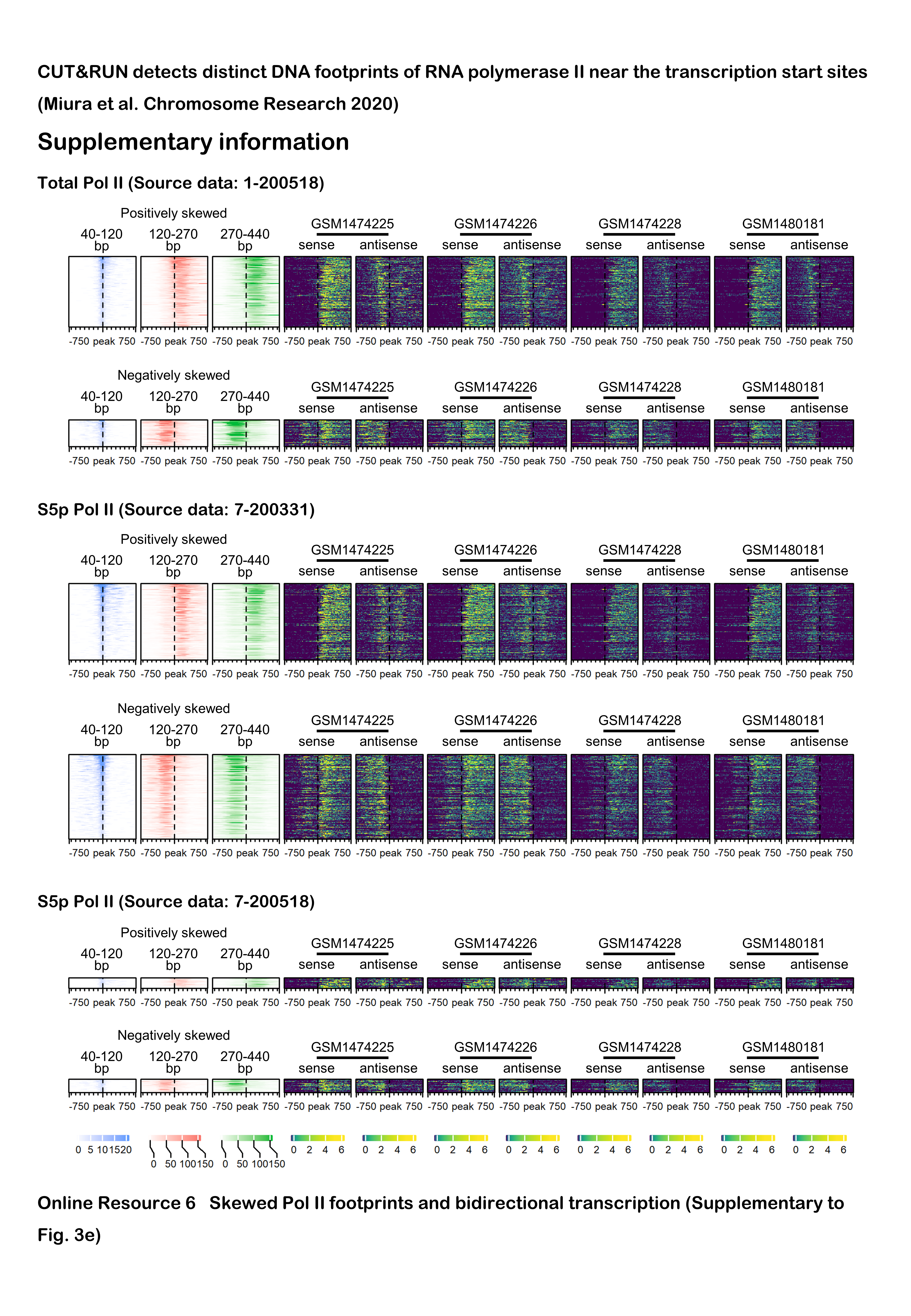

Supplement: Supplementary file 12 — High Resolution Image (TIF 11667 kb) [file 10577_2020_9643_MOESM6_ESM.tif]
